# Cell therapy could be a potential way to improve lipoprotein lipase deficiency

**DOI:** 10.1186/s12944-017-0577-4

**Published:** 2017-10-02

**Authors:** Wenjing Wu, Yajun Yin, Jie Zhong, Yongjia Peng, Shuncai Li, Libin Zheng, Hong Cao, Jin Zhang

**Affiliations:** 1 College of Biological and Chemical Science and Engineering, Jiaxing University, Lianglin Campus,118 Jiahang Road, Jiaxing, 314001 China; 2 College of life science and biotechnology, Hebei Normal University of Science and Technology, Qinhuangdao, 066004 China

**Keywords:** Lipoprotein lipase deficiency, Cell therapy, Retrovirus, Adipocytes

## Abstract

**Background:**

Lipoprotein lipase (LPL) deficiency is an autosomal recessive genetic disorder characterized by extreme hypertriglyceridemia, with no cure presently available. The purpose of this study was to test the possibility of using cell therapy to alleviate LPL deficiency.

**Methods:**

The LPL coding sequence was cloned into the MSCV retrovirus vector, after which MSCV-hLPL and MSCV (empty construct without LPL coding sequence) virion suspensions were made using the calcium chloride method. A muscle cell line (C2C12), kidney cell line (HEK293T) and pre-adipocyte cell line (3 T3-L1) were transfected with the virus in order to express recombinant LPL in vitro. Finally, each transfected cell line was injected subcutaneously into nude mice to identify the cell type which could secret recombinant LPL in vivo. Control cells were transfected with the MSCV empty vector. LPL activity was analyzed using a radioimmunoassay.

**Results:**

After virus infection, the LPL activity at the cell surface of each cell type was significantly higher than in the control cells, which indicates that all three cell types can be used to generate functional LPL. The transfected cells were injected subcutaneously into nude mice, and the LPL activity of the nearby muscle tissue at the injection site in mice injected with 3 T3-L1 cells was more than 5 times higher at the injection sites than at non-injected control sites. The other two types of cells did not show this trend.

**Conclusion:**

The subcutaneous injection of adipocytes overexpressing LPL can improve the LPL activity of the adjacent tissue of nude mice. This is a ground-breaking preliminary study for the treatment of LPL deficiency, and lays a good foundation for using cell therapy to correct LPL deficiency.

## Background

Lipoprotein lipase (LPL), which is the rate-limiting enzyme of lipoprotein tryglyceride metabolism, is synthesized and secreted by adipocytes, myocardial cells, skeletal muscle cells and kidney cells [1, 2]. After secretion, LPL is transported to the capillary endothelial cells and binds to the cell surface with the aid of heparan sulfate proteoglycans (HSPG) [3]. Apolipoprotein CII in the active, non-covalently bound homodimer form, activates LPL to hydrolyze chylomicrons (CM) in the blood and triglycerides (TG) in very-low-density-lipoprotein (VLDL), resulting in the provision of fatty acids and monoacylglycerols to the body [4, 5]. The most common reason for abnormal activity of plasma LPL is a genetic defect in the LPL gene [6–8]. LPL deficiency is an autosomal recessive genetic disorder characterized by extreme hypertriglyceridemia (plasma triglyceride levels of 11 mmol/L, with less than 1.7 mmol/L being normal), plasma samples having a milky consistency and color, and triglyceride levels nearly 10 times higher than normal [9, 10]. When eating a normal diet, the patients display ongoing abdominal pain, enlarged liver and spleen, acute pancreatitis, lipid retinitis, eruptive xanthoma and fasting chylomicronemia [11]. In cases of childhood onset, dietary restrictions are significant and quality of life is low [12]. To date, there is no known cure for LPL deficiency [13] .

This study set out to develop a strategy for the treatment of LPL deficiency using induced pluripotent stem cells. The feasibility of this idea was verified in a mouse model in three steps. Firstly, fibroblasts were isolated from the LPL-deficient model mice and modified using induced pluripotent stem cell treatment technology through the introduction of 2–4 different transcription factors for reprogramming [14] in order to obtain LPL-deficient stem cells. Secondly, the LPL genotype of the stem cells was corrected using an LPL cassette as the gene treatment carrier. Finally, the resulting stem cells were differentiated to yield highly efficient actively LPL-producing tissue cells, and were transplanted back into the mice in vivo, with the expectation that they could cure the LPL deficiency. This method avoids the problems of allograft immune rejection and has good prospects for clinical application [14].

Presently, the technology for reprogramming fibroblasts to produce induced pluripotent stem cells is rapidly maturing [15], and commercial test kits are available. Therefore, two main points left to be solved for this project were the need to construct the genotype correction gene therapy vector for the LPL-deficient stem cells, and secondly to induce the stem cells to differentiate into the kinds of tissues with efficient LPL secretion in vivo, that are also convenient for clinical transplantation.

This paper reports the screening of gene vector constructs and ideal transplanting tissues, resulting in the successful construction of the retroviral vector MSCV-hLPL, which led to the preliminary confirmation that induced adipocytes can carry foreign LPL genes and can stably secrete LPL to the surrounding tissue after transplantation.

## Methods

### Vectors, bacterial strains and experimental mice

The retroviral vector MSCV-IRES-GFP (http://www.addgene.org/20672/) and its virion-packaging auxiliary plasmid pCL-Eco (http://www.addgene.org/12371/) were kindly provided by Professor Dennis Roop from the University of Colorado, Denver Campus, USA. The vector psk-hLPL, encoding human LPL cDNA, was kindly provided by Professor Robert Eckel from the same university. *E. Coli* Top10 was purchased from Invitrogen, USA. Mouse preadipocyte cell line3 T3-L1, mouse myoblast cell line C2C12 and human embryonic kidney cell line HEK293T were maintained by our laboratory.

### Enzymes and biochemical reagents

High-fidelity Phusion DNA polymerase and all restriction enzymes were purchased from the New England Biotechnology company, USA. Ampicillin, fetal bovine serum, and DMEM culture medium were purchased from Sigma-Aldrich, USA. Ligase T4 DNA was purchased from the Invitrogen, USA.

The DNA extraction kit, plasmid mini-preparation kit and large quantity extraction kit were purchased from Qiagen, Germany.

### Construction of the MSCV-hLPL vector

We used pSK-hLPL as template to design the cloning primers F1 (containing a stop codon) and R1, containing *Xho* I and *Eco*R I restriction sites (Table 1). The fragment was amplified via PCR. The resulting product was purified, digested with the two enzymes overnight, and inserted into the corresponding sites of the MSCV vector. The primers F2 and R2 (Table 1), which bind upstream and downstream of the MSCV cloning site, were used for colony PCR and sequencing.

**Table 1:** Primers used in study

Primer name	Primer sequence (5′–3′)
hLPL-F1	ATCCGCTCGAGATGGAGAGCAAAGCCCTGC
hLPL-R1	CCGGAATTCTCAGCCTGACTTCTTATTCAGAGAC
MSCV-F2	CCCTTGAACCTCCTCGTTCGACC
MSCV-R2	CATATAGACAAACGCACACCGGC

The underlined sections indicate the restriction sites of Xhol and EcoRI

### Virion packaging, infection and analysis of recombinant hLPL activity

The Phoenix-ECO cell line was used to produce a viral suspension using the calcium chloride method [16]. The 3 T3-L1, C2C12 and HEK293T cell lines were all grown in DMEM (Gibco, China) comprising 10% FBS (fetal bovine serum; Gibco, the USA). When the cells reached 70% confluence in the petri dish, 0.1% of a 1% poloyborne solution (Gibco, the USA) was added, followed by the virus suspension at a ratio of 5:1 (nutrient solution: virus suspension), and mixed gently. The medium was changed 8 h after infection. The following day, the procedure was repeated, followed by media changes every 8 h. After 48 h of culture, the cells were harvested and their LPL activity was analyzed using the radioimmunoassay method [16, 17]. The control cells were transfected with virions containing the empty construct MSCV.

### Injection into nude mice and analysis of recombinant hLPL activity

The MSCV-hLPL-infected C2C12, 3 T3-L1 and HEK293T cells were separately harvested, resuspended in PBS, and the cell concentration adjusted to 10^7^ mL^−1^. The resulting cell suspension was injected subcutaneously into the hips of nude mice, into both the right and left side, administering 1 mL (10^7^ cells) at each site. In total, nine nude mice were used in this study. Three mice were injected with MSCV-infected cells as the blank therapy group, and were named control mouse 1 (injected with C2C12-MSCV), control mouse 2 (injected with 3 T3-L1-MSCV) and control mouse 3 (injected with HEK293T-MSCV). Six mice were injected with MSCV-hLPL-infected cells as the therapy group, and were named as therapy mouse 1 & 2 (injected with C2C12-MSCV-hLPL), therapy mouse 3 & 4 (injected with 3 T3-L1-MSCV-hLPL), and therapy mouse 5 & 6 (injected with HEK293T-MSCV-hLPL). 12 to 20 days after injection, the nude mice were sacrificed in batches, followed by analysis of LPL activity in the muscle and skin injection sites using the radioimmunoassay method. Skin and muscle of each nude mouse at the non-injection site (behind the front legs) was compared to the injection site, and the differences between the therapy group and blank therapy group were analyzed.

### Statistical analysis

The experimental data were obtained from at least three independent experiments and expressed as means ± SEM. The data were analyzed using one-way or two-way ANOVA. Differences were considered statistically significant at *P* < 0.05.

## Results

### Successful construction of the MSCV-hLPL vector

**Fig. 1:**
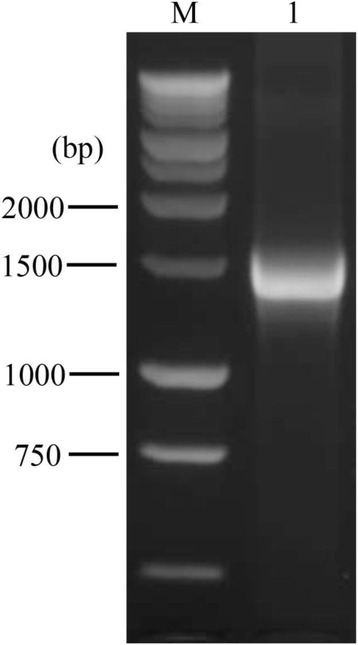
PCR amplification of human LPL coding sequence

The 1.4 kb coding sequence of human LPL (hLPL) was cloned (Fig. 1) from the plasmid pSK-hLPL using the primers hLPL-F1 and hLPL-F2 [18] (Table 1). The coding sequence was inserted into the retroviral vector MSCV (http://www.addgene.org/20672/) via its *EcoR* I and *Xho* I restriction enzyme cutting sites. The recombinant construct was subsequently used to transform *E. coli* strain Top 10 for amplification and verification by sequencing.

### The muscle cell line C2C12, Preadipocyte cell line 3 T3-L1 and kidney cell line HEK293T efficiently produced LPL in vitro

**Fig. 2:**
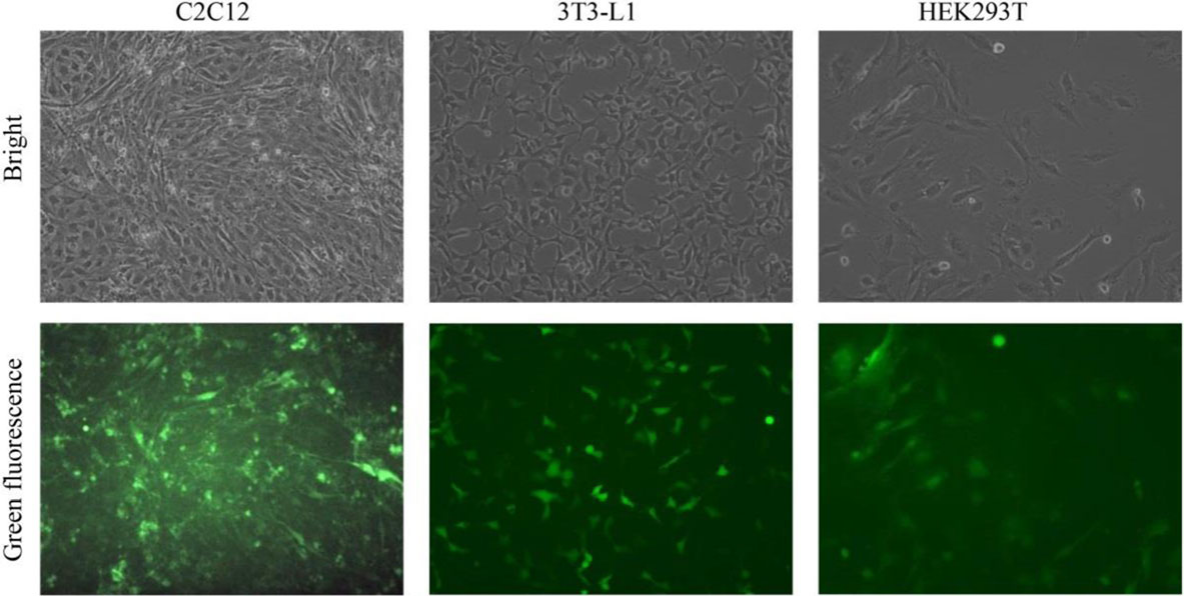
MSVC-hLPL transfection ratio analyses. The cells with expression of EGFP which was green under the fluorescence microscope were regarded as transfected cells. The transfection ratio was estimated by the percentage of transfected cells

**Fig. 3:**
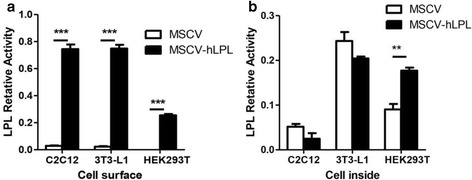
LPL activity assay of three cell lines transfected with MSCV-hLPL (4 days after infection) (**a**) The LPL activity of three cell lines﻿ at the cell surface. **b **The LPL activity of three cell lines in the cell inside

Using the calcium chloride transfection method, the MSCV-hLPL virion suspension was obtained using the retro-viral packaging system Phoenix-ECO developed by Stanford University [19]. The virus was able to infect the muscle cells (C2C12), kidney cells (HEK293T) and preadipocyte (3 T3-L1) effectively (Fig. 2). The intracellular and cell-surface LPL activity was analyzed by the radioimmunoassay method [16, 17]. Before infection, C2C12 showed similar LPL activity both inside the cells and on the cell surface; 3T3L1 showed more intracellular LPL activity than at the cell surface, while HEK293T only showed intracellular LPL activity. After infection with the MSCV-hLPL virus, the LPL activity of each cell line increased dramatically at the cell surface, compared with the respective control groups transfected with the empty control virus (Fig. 3). The surfaces of 3 T3-L1 and C2C12 cells showed similar LPL activities, which were about two times higher than what was observed at the HEK293T surface. After transfection, LPL activity increased 36 times at the surface of 3 T3-L1, and 12 times at the surface of C2C12 cells. Thus, 3 T3-L1, C2C12 and HEK293T cells were all able to express recombinant LPL after transfection with the MSCV-hLPL virus.

**Fig. 4:**
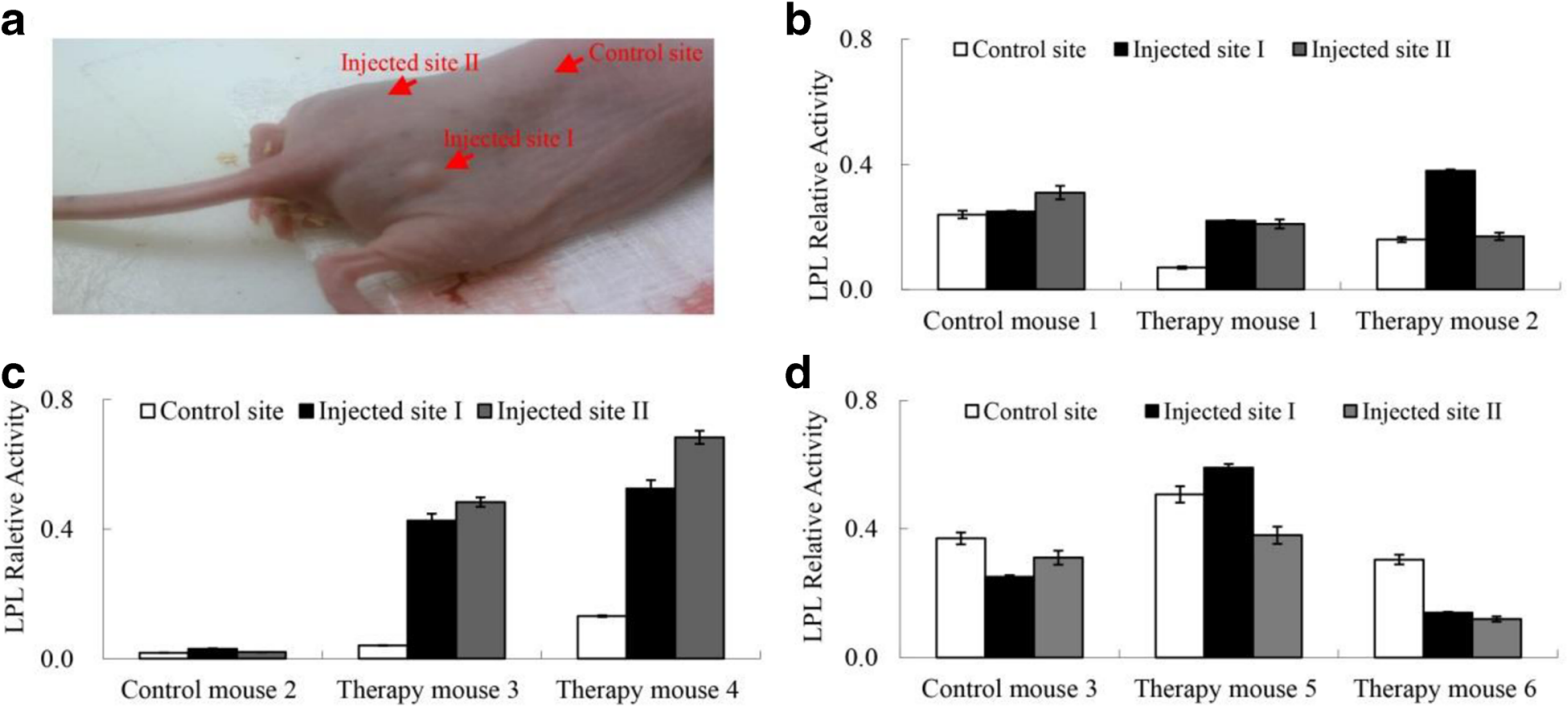
LPL activity assay of muscle tissue under the injection site of nude mice. **a** Indication of the injection sites and the control site(Each mouse was injected two sites (left and right hip). The control site was back muscle tissues of the same mouse two inches away from each injection site.). **b** The mice injected with C2C12. **c** The mice injected with 3 T3-L1. **d** The mice injected with HEK293. Note. Blank therapy mice were injected with the corresponding cell line transfected with the empty-vector control virus MSCV; Therapy group mice were injected with cell lines which were transfected with the LPL expression virus MSCV-hLPL; Each mouse was injected in two sites (left and right); The control site was part of the non-injected tissue at a distance of 2 cm from the injection site

### 3 T3-L1 cells can be used to secrete recombinant LPL in vivo

Each transfected cell line was injected subcutaneously into the nude mice. The quantity of the cells was approximately 10^7^ at each injection site. Each cell type was injected into 3 nude mice at two different injection sites (left and right hip, respectively; Fig. 4a) in each mouse. One mouse acted as the blank therapy group and was injected with the cells transfected with the empty MSCV virus. The remaining two mice acted as the therapy group and were injected with the cells transfected with the MSCV-hLPL virus. Blood extraction was performed every 3 to 5 days. On day 12, the HEK293T-injected mouse was sacrificed, on day 16 the C2C12-injected mouse was sacrificed, and the 3 T3-L1-injected mouse was sacrificed on day 20.

Analysis of LPL activity in blood samples did not reveal any differences between the therapy group and the blank therapy group (data not shown). Subsequently, LPL activity of the tissues around the injection site (lower muscle tissue at the injection sites) was analyzed, and compared with tissues two cm away from each injection site as a control (Fig. 4a). The lower muscle tissue in the therapy group injected with 3 T3-L1 showed higher LPL activity than the control tissues, while the blank therapy group did not show this trend (Fig. 4c). >LPL activity increased more than five-fold in the lower muscle tissues with the injection of 3 T3-L1. However, the injection of C2C12 and HEK293T did not significantly affect the LPL activity in any of the mice (Fig. 4b & d).

## Discussion

In 2006, the Japanese scholar Shinya Yamanaka published on the induced pluripotent stem cell (iPSC) technology for the first time [20]. In recent years, this technology has attracted the attention of many scholars and business groups because of its promising applications in the field of regenerative medicine [21–24]. Although iPSC technology still has some problems, such as low induction rates, incomplete reprogramming, and potential biological safety issues arising from the necessary use of viral vectors [25], sooner or later these problems will be overcome through technological development. However, the proper ways to apply iPSC technology in clinical medicine still require serious thinking. Tissues and organs are primarily expected to be cultured in vitro through iPSC technology, to be used for organ transplantation, but very little progress was made in this area in the past years. This project selected LPL deficiency as the subject of study even though the incidence of this disease is very low - only one in a million. However, the study nevertheless has dual significance: first, the patients’ quality of life is seriously affected by this disease, with patients requiring more social care and assistance; second, LPL deficiency is a useful model of a disease with single-gene inheritance, which makes it significant for scientific research. If the use of iPSC technology can overcome LPL deficiency, the same research strategy can be applied to other single-gene defects.

This project therefore provides a concept of cell therapy for the treatment of LPL deficiency. In the first step, an MSCV vector comprising a recombinant LPL coding sequence was constructed, and it exhibited high infection rates. MSCV is a modified retroviral vector (http://www.addgene.org/18770/). This vector can not only infect most cell lines and primary cells, but it can also infect stem cells, which was very much in line with the requirements of this project.

This research also proved that MSCV-hLPL can efficiently infect a variety of cells cultured in vitro. However, the safe usage of retroviral vectors is controversial [26]. Nevertheless, since the primary task of this project was to prove that the genetic defect causing LPL deficiency is curable, these problems can be left for future research on cell therapy, which will lead to the development of a safe and efficient vector.

LPL expression can be detected in many different tissues in mice, but the active LPL in vivo is mainly provided by adipose tissue, skeletal muscle tissue, myocardial tissue and kidney tissue [4]. Therefore, muscle cells (C2C12 cell line), kidney cells (HEK293T) and preadipocytes (3 T3-L1) were selected for the in vitro study of the expression of recombinant LPL. To our best knowledge, the use of in vitro cultured cells to secrete active LPL has not been reported previously. Each cell line used in this study can not only produce lipoprotein lipase after infection with the MSCV-hLPL virus, but can also efficiently secrete LPL to the cell surface, which is a necessary precondition for proper lipoprotein lipase function [3]. This confirms that these three cell lines can act as hosts to produce recombinant LPL in vitro. This study is to our best knowledge the first one reporting on the secretion of active recombinant LPL by C2C12, 3 T3-L1 and HEK293T cells transformed using retroviral vectors.

Finally, the use of an in vivo mouse model proved that 3 T3-L1 can secrete active LPL into nearby muscle tissue, improving its LPL activity. Because the mice used in this study are able to produce LPL, the amount of recombinant LPL produced through cell injection was negligible compared to the amount of LPL produced by the mouse itself. It is therefore not surprising that blood analysis of the mice revealed no significant differences of LPL activity between the blank control group and the therapy group. Consequently, we used a dual-control research methodology to compare the injection site with a non-injection site in each individual nude mouse, thereby ruling out the effects of individual differences between the mice on the experimental results. Moreover, we used a blank therapy group as a negative control. An analysis of the LPL activity at the injection site and non-injection site of the blank therapy mice was used to rule out any effect of the injected cells themselves on LPL secretion in the mice. Through this dual-control design, the secretion of recombinant LPL by the recombinant cells into nearby muscle tissue was detected. The injection of the modified 3 T3-L1 cells improved the LPL activity more than five-fold in the lower muscle tissues, which represented the best result. This is a favorable result because adipocytes are one of the easiest cell types to transplant [10].

Based on the results of this research project, fibroblasts from the body of an LPL-deficient patient can be analyzed, reprogrammed into pluripotent stem cells [27, 28], the MSCV-hLPL virus can be used to correct the LPL deficient genotype, and the thus modified cells can be differentiated into pre-adipocytes. Finally, the resulting LPL-producing adipocytes can be transplanted back into the patient’s body with the hope of improving or curing LPL deficiency.

## Conclusion

This study used a mouse model to demonstrate that recombinant adipocytes modified to express LPL can alleviate lipoprotein lipase deficiency. The protocols for stem cell differentiation into adipocytes are very mature now [29]. For transplantation, it is easier to work with fatty tissue than with other tissues, as long as the transplanted tissue is implanted subcutaneously into the patients. Therefore, this work represents a pioneering attempt in this field, laying a solid foundation for the use of stem cells for the development of therapies for lipoprotein lipase deficiency.

## Abbreviations

apoB: Apolipoprotein B
CM: Chylomicron
HDL: High density lipoprotein
HSPG: Heparan sulfate proteoglycan
iPSC: Induced pluripotent stem cells
LPL: Lipoprotein lipase
TG: Triacylglycerol
VLDL: Very-low-density lipoprotein
